# Thermodynamic and kinetic analysis of the response surface method for phenol removal from aqueous solution using graphene oxide-polyacrylonitrile nanofiber mats

**DOI:** 10.1038/s41598-024-53572-5

**Published:** 2024-02-12

**Authors:** Basant Yousri Eweida, Asmaa M. Abd El-Aziz, Azza El-Maghraby, Eman Serag

**Affiliations:** 1 Modeling and Simulation Research Department, Advanced Technology and New Materials Research Institute, City of Scientific Research and Technological Applications, Borg El-Arab, Alexandria, Egypt; 2 Fabrication Technology Research Department, Advanced Technology and New Materials Research Institute, City of Scientific Research and Technological Applications (SRTA-City), Borg El-Arab, Alexandria, Egypt; 3 Marine Pollution Department, Environmental Division, National Institute of Oceanography and Fisheries (NIOF), Kayet Bey, Elanfoushy, Alexandria, Egypt

**Keywords:** Graphene oxide, Poly acrylonitrile, Nanofiber composite, Phenol removal, Kinetics, Thermodynamics, Chemistry, Nanoscience and technology

## Abstract

Phenolic compound even at low concentrations, are considered to be priority pollutants due to their significant toxicity. Electrospinning was used to create a polyacrylonitril (PAN) nanofiber, which was then impregnated with graphene oxide (GO). After a preliminary investigation into the electrospinning parameters (e.g., using various voltages and polymer concentrations), the electrospun nanofibres were tuned, this study evaluated the effectiveness of these materials in removing phenolic compounds from wastewater through adsorption. Scanning electron microscopy (SEM) and Fourier transform infrared spectroscopy (FTIR) were used to analyze the synthesized nanofiber mats. The scanning electron microscopy (SEM) analysis revealed that the structure of nanofiber mats was altered by the addition of graphene oxide (GO) in different ratios. Specifically, the surface of the fibres exhibited increased roughness, and the diameter of the fibres also experienced an increase. The average diameter of the fibres was measured to be (134.9 ± 21.43 nm) for the PAN/2.5% GO composite and (198 ± 33.94 nm) for the PAN/5% GO composite. FTIR spectra of the PAN/GO nanocomposites nanofiber displayed distinct peaks associated with graphene oxide (GO). These included a wide peak at 3400 cm^−1^, related to the presence of hydroxyl (O–H) groups, as well as peaks on 1600 as well as 1000 cm^−1^, which indicated the existence of epoxy groups. In this study response surface methodology (RSM) was implemented. To enhance the efficiency of removing substances, it is necessary to optimise parameters such as pH, contact time, and dosage of the adsorbent. The optimum pH for removing phenol via all nanofiber mats was determined to be 7, while at a dose of 2 mg dose adsorbents maximum removals for pure PAN, PAN/2.5 GO, and PAN/5 GO were 61.3941, 77.2118, and 92.76139%, respectively. All the adsorbents obey Langmuir isotherm model, and the empirical adsorption findings were fitted with the second-order model kinetically, also non-linear Elovich model. The maximal monolayer adsorption capacities for PAN, PAN/2.5 GO, and PAN/5 GO were found to be 57.4, 66.18, and 69.7 mg/g, respectively. Thermodynamic studies discovered that the adsorption of phenol on all adsorbents nanofiber mats was exothermic, the adsorption of phenol on nanofiber mats decreases as the temperature increases. All the adsorbents exhibit negative enthalpy and entropy. The PAN/GO composite's superior phenol removal suggested that it could be used as a latent adsorbent for efficient phenol removal from water and wastewater streams.

## Introduction

The escalating demand for water to support agricultural production, maintain industrial operations, and accommodate the expanding human population has led to intensified competition for scarce freshwater resources^1,2^. The utilisation of wastewater is increasingly being recognised as a viable approach to address the escalating demands for water^3^. The proliferation of nonbiodegradable organic pollutants within wastewater, coupled with the absence of efficient methods for their removal, presents a significant challenge to the reutilization of such wastewater. Phenols and their derivatives, originating from industrial activities and agricultural runoff contaminated with pesticides, constitute the predominant organic pollutants detected in wastewater^4^. Phenolic compounds exhibit a prolonged environmental persistence, leading to their accumulation and consequent toxicological impacts on both human and animal populations. Even at minimal levels, these substances exhibit significant toxicity towards human beings^5^. Phenolic compounds pose significant risks to human health, as they have been found to cause tissue erosion, liver and kidney damage, elevated blood pressure, and central nervous system paralysis^6^. The natural degradation of phenol in aqueous environments occurs, provided that the concentration remains below a threshold that would substantially hinder microbial activity. Moreover, the rates of phenol degradation are influenced by the coexistence of various organic or inorganic pollutants in water, alongside phenol^7^. The adsorption method is widely documented as a highly effective and economical technique for eliminating pollutants from water, simplified process, and minimal energy consumption. This assertion holds particular significance in the context of non-degradable micropollutants^8^.Graphene oxide is derived from graphene, Graphene oxide (GO) has garnered considerable interest in recent times owing to its noteworthy physical and chemical attributes, encompassing optical, catalytic, and mechanical properties. It is a two-dimensional material that consists of carbon atoms arranged in a hexagonal lattice. Furthermore, graphene oxide (GO) exhibits adsorbent properties towards water pollutants, as well as heavy metals, organic pollutants in addition to dyes. The reason for this is the extensive surface area and the existence of functional groups that can cooperate with pollutants. Thereby facilitating the adsorption processes. In addition, graphene oxide (GO) exhibits a notable efficacy in the removal of organic pollutants containing benzene rings due to its strong affinity towards such compounds through the π-π interaction mechanism^9^. Nevertheless, the occurrence of carboxyl and hydroxyl functional groups on the surface of graphene oxide (GO) engenders its buoyancy in aqueous environments, thereby presenting a potential hazard to both ecological systems and human well-being^10^. Integrating graphene oxide (GO) into a polymeric matrix has been found to yield distinctive properties that have potential applications in various fields, including water treatment^11^. The utilisation of Polyacrylonitrile nanofibers (PAN) matsfor water pollutants adsorption has been observed in recent studies. Due to their substantial specific surface areas, elevated porosity, and finely adjustable surface properties^12^. Nevertheless, the mechanical properties of PAN nanofiber membranes are suboptimal, necessitating their enhancement through the incorporation of additional polymer or inorganic filler nanomaterials, such as graphene oxide (GO)^13^. The intent of this work was to inspect the development of innovative membranes consisting of electrospun nanofibers made from a combination of polyacrylonitrile and graphene oxide (PAN/GO). The primary focus was to incorporate the graphene oxide within the fibre structure, with the intention of utilising this technology for environmentally friendly adsorption Insofar as we are aware, the utilisation of these composites in the phenol removal percentage (%) from aqueous solutions has not been sight seen through the application of RSM and the adsorption process. RSMis a widely utilised statistical model in experimental design for the purpose of optimising different processes through the application of a quadratic polynomial model. The advantage of Response Surface Methodology (RSM) lies in its capacity to reduce the expenses associated with intricate analysis techniques by minimising the quantity of experiments needed. The execution of this approach can be achieved by employing either the Box-Behnken Design (BBD) or the central composite design (CCD) methodologies. The main intention of this work was to optimise the efficacy of phenol removal from an aqueous solution via investigating various factors, such as concentration (ppm), dosage (mg), and duration (min). The Box-Behnken Design (BBD) was employed for this purpose.

### Materials

Graphite Flakes (acid treated 99%, Asbury Carbons), potassium permanganate (99%, RFCL), hydrogen peroxide (30% wt, Emplura), Polyacrylonitrile PAN (MWT = 150,000 g/mol, d = 1.184 g/mL) at 25° C, N,N dimethylformamide (C_3_H_7_NO), and Phenol were purchased from Sigma-Aldrich Co. (USA), sodium nitrate (98%, Nice Chemicals), and sulphuric acid (98%, ACS) from thermos FisherScientific.

### Methods

#### Synthesis of graphene oxide nanosheets

The production of graphene oxide nanosheets was employed by modified Hummer's method. To put it simply, the mixture of 1 g of graphite and 1/2 g of NaNO_3_ was mixed thoroughly. And subsequently, with constant stirring, 23 millilitres of concentrated H_2_SO_4_ were added, Additionally, in order to avoid overheating, the temperature of reaction was sustained below 20 °C. After a vigorous stirring for one hour, 3 g of KMnO_4_ were added to the mixture. Before adding 500 ml of distilled water, the reaction mixture was stirred for an additional 12 h at a temperature of approximately 35 °C. Running the reaction mixture at 98 °C for 40 min followed addition of water. Fifty millilitres of 30% H_2_O_2_ was added once the reaction mixture had cooled. After centrifugation, the solution was rinsed with an aqueous 1N HCl solution to extract any remaining metal ions. In order to bring the solution's pH up to 6.5, it was rinsed again with double-distilled water. We then subjected the resulting solid of yellow–brown graphene oxide to dry under vacuum for 24 h^14^.

#### Preparation of polymeric nanofiberous mats

A 5 weight % PAN solution was used to prepare the polymeric nanofibers (control sample), and to prepare PAN/GO nanofiber composites the (GO-NP) were added to the polymeric solutions with different concentrations (2.5, and 5) wt%. It was necessary to use a syringe pump to continuously inject the polymer solutions into a syringe needle at a rate of 0.5 mL.h^−1^. A flat aluminium foil collector, 10 cm from the needle, was used to electrospun PAN solutions at a constant voltage of 20 kV. *The characterization techniques have been shown in supplementary data.*

#### Experimental design

The optimisation of phenol removal percentage from an aqueous solution using PAN/ GO was conducted through the implementation of the Box-Behnken design^15^.

To examine the effects of three independent variables—concentration (ppm), dose (mg), and time (min)—that were each varied at three levels (− 1, 0, and 1), a Box-Behnken design (BBD) was utilised^16,17^.

Where, fifteen experimental runs were conducted to assess the impact of three variables on the percentage of phenol removed from an aqueous solution: concentration (ppm), dose (mg), and time (min). The experimental variables were X1 (100, 300, 500), X2 (0.5, 2, 5), and X3 (0, 30, 60). The quadratic equation was used to calculate the correlation between the response and independent variables after the experiments were conducted has been shown in Eq. (S1).

#### Phenol removal tests

The adsorption batch experiments were conducted by utilising 5 mg of the nanofiber mats immersed in a phenol solution with an initial concentration of 100 mg/l. The experiments were demeanor at room temperature and pH 7, while subjecting the solution to agitation at a speed of 200 rpm for a duration of 120 min. Utilizing a UV spectrophotometer (Shimadzu, UV240, Japan) with a 285 nm wavelength, the concentrations of phenol were determined. Adsorption experiments were carried out at different pH levels to identify the optimal adsorption conditions. The pH was determined using a benchtop pH metre (A0057419, Hanna). The adsorption batch experiment was performed at the optimum pH, and aliquots of the phenol solution were taken out at regular intervals to study the kinetic parameters. In order to calculate the equilibrium assay, various concentrations of phenol solution, ranging from 100 to 500 ppm, were also absorbed. The experiments were conducted in duplicate (n = 2), and equation (S2) was utilised to calculate the adsorption equilibrium (qe).

#### Adsorption isotherms

To learn how molecules interact with one another in the equilibrium state of an adsorption process, isotherm studies were conducted^19^. To comprehend the phenol's adsorption behaviour on nanofiber mats, linear Langmuir and Freundlich isotherm models were developed, equation (S3, S4).

#### Kinetics Studies

To gain understanding of the adsorption mechanism and rates, theoretical modelling of sorption kinetics was carried out. The kinetic data was analysed using various models, including the linear kinetic pseudo-first-order, pseudo-second-order, nonlinear kinetic intra-particle diffusion, and Elovich models, equation (S5, S6, S7, S8).

#### Adsorption thermodynamics model

The thermodynamic properties of phenol, including the change in Gibbs free energy (ΔGº), change in enthalpy (ΔHº), and change in entropy (ΔSº), are determined using the equation. Equations (S9, S10) and (S11)^22^.

## Results and discussion

**Figure 1:**
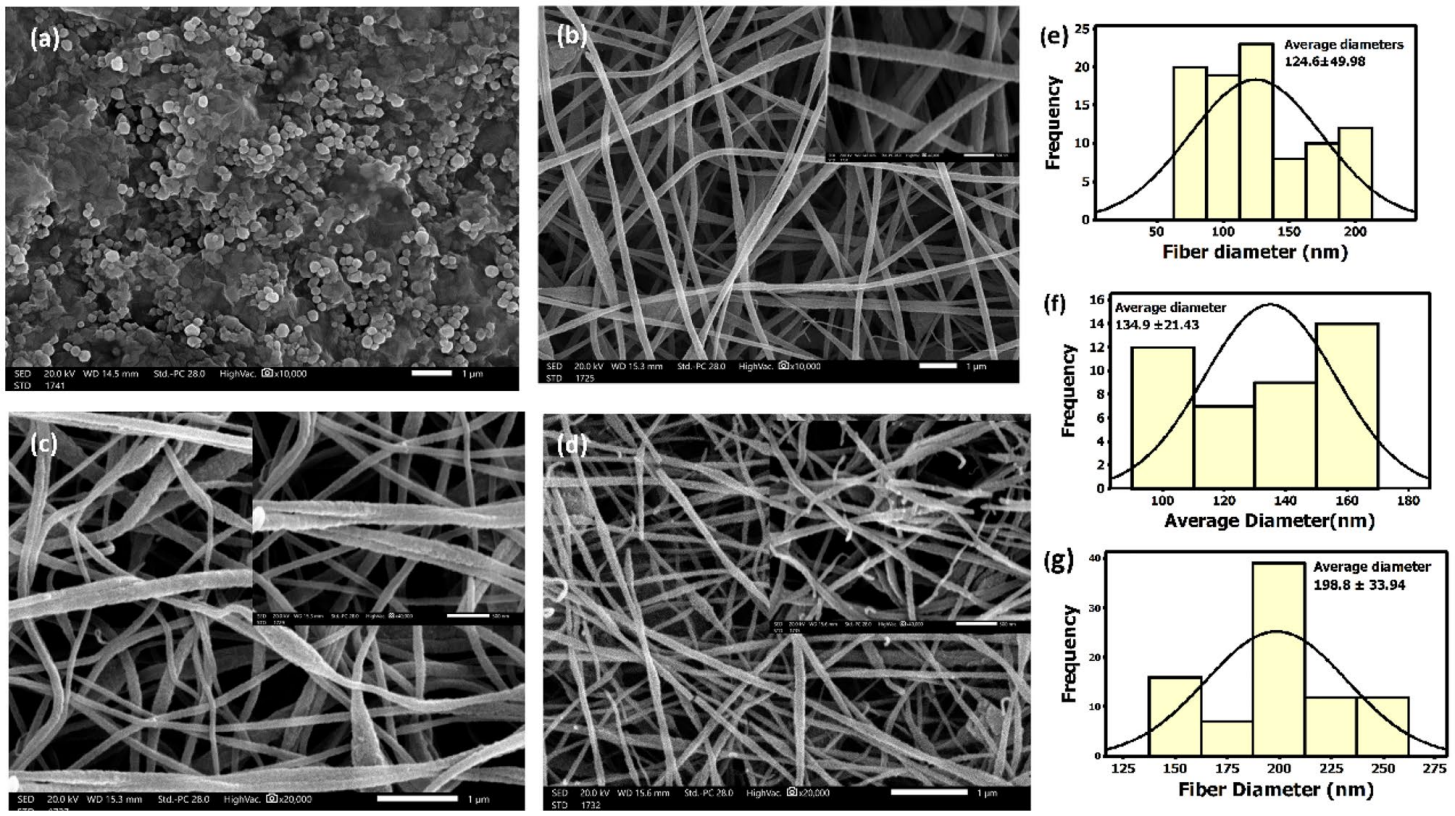
SEM images for (**a**) the synthesized graphene oxide (**b**) pure PAN nanofibers mats (**c**) PAN/2.5 GO nanofibers, (**d**) PAN/5 GO nanofibers, (**e**), (**f**), and (**g**) represent the average diameters of pure PAN nanofibers mats, PAN/2.5 wt% GO nanofibers, PAN/5 wt% GO nanofibers, respectively.

The average diameter distribution in addition to the surface morphology of the synthesized GO, pure PAN nanofiber mats, and PAN/GO nanofibers mats composites (Fig. 1). Scanning electron microscope images revealed that different hybrid nanostructures of GO, nanoparticles of various sizes (111–236 nm), which were synthesized directly from graphite, as well as exfoliated GO sheets with no overlapping or aggregation, and this GO morphology was also achieved by previous work^23^. Figure 1b shows that pure PAN nanofibers mats were synthesized with a smooth surface and bead-free with an average fiber diameter of (124 ± 49.98 nm), whereas by adding GO in different proportions to the electrospun, the surface of the fibers became rough and the diameter increased with an average diameter of (134.9 ± 21.43 nm) for PAN/2.5 wt% GO and (198 ± 33.94 nm) for PAN/5 wt% GO. This is due to GO agglomeration in the fibers^24^. Our results have been compared with the previous work as shown in Table 1.

**Table 1:** Comparison between the previous work to the our recent prepared nanofiberous mats.

PAN concentration & GO concentration	Eectrospinning parameters	Results	Reference
PAN (5 wt%) & GO (2.5, 5) wt%	The tip-to-collector distance being 10 cm, the voltage being 20 kV, and the ejection speed being 0.5 mL/h	PAN (124 ± 49.98 nm)	This work
		PAN/2.5 GO (134.9 ± 21.43 nm)	
		PAN/5GO (198 ± 33.94 nm)	
GO (2–30 wt%)	The tip-to-collector distance being 15 cm, the voltage being 15 kV, and the ejection speed being 0.8 mL/h	The dispersion and average diameter of the individual nanofiber strands seem to thicken with increasing applied GO content	^30^
GO (1 wt%) into DMF/deionized water mixed solvent, (10 wt%) PAN with GO (1.98 wt%)	The tip-to-collector distance being 20 cm, the voltage being 15 kV, and the ejection speed being 0.5 mL/h	PAN nanofibers had an average diameter of 475 ± 53 nm, a smooth surface, and a homogeneous diameter distribution	^31^
		The resulting composite PAN nanofibers had a complicated porous morphology and a rough surface with average diameter 1356 ± 267 nm	
PAN (10 wt%) Various concentrations of GO (0.05, 0.5, 1, and 1.5 wt%)	The tip-to-collector distance being 15 cm, the voltage being 25 kV, and the ejection speed being 0.5 mL/h	average fiber diameters are 465, 383, 326, 288, and 221 nm, for samples (0, 0.05, 0.5, 1.0, 1.5) GO wt%	^32^

**Figure 2:**
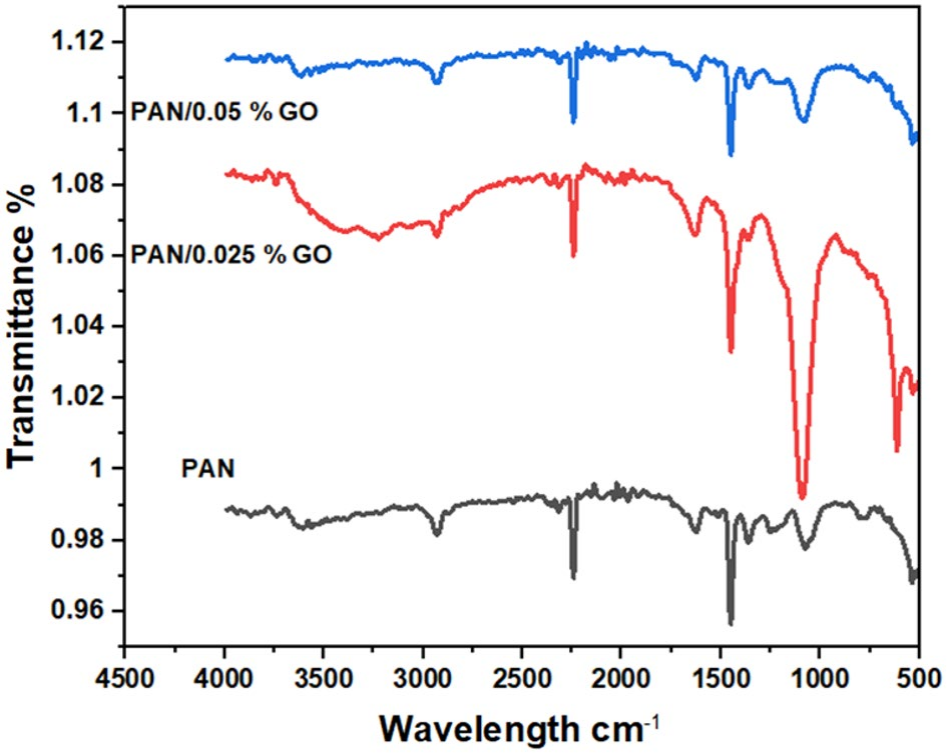
FTIR pattern for the pure PAN, and PAN/GO nanofibers mats composites.

Figure 2 shows the FTIR spectra of the synthesized GO, pure PAN and PAN/GO nanofiber composites. The broad band in the FTIR spectrum of GO at 3500 cm^−1^was associated with the C–OH of the carboxyl group^25^, the vibration at 1600–1650 cm^−1^ represented the stretching of the C=O carbonyl group^26^, the peak at 1500 cm^−1^ represented the sp^2^ hybrided C=C, and the band at 1200 cm^−1^ was associated with the C–O–C of the epoxide group^27^. Pure PAN spectra showed a vibration band at 2926 cm^−1^^28^, and 1454 cm^−1^ are assigned to C–H belong to methyl group of isoprene. The PAN/GO nanocomposites nanofiber's spectra revealed typical peaks for GO, a broad peak at 3400 for the O–H group, and peaks at 1600 and 1000 cm^−1^ that are assigned to epoxy groups^29^.

### RSM analysis for optimization of removal percent (%) of phenol

The RSM study analyzed the experiments using the standard Box-Behnken factorial design (BBD). The first single parameter tests were completed in order to examine the levels of the variables influencing the percent (%) of phenol elimination. Following the selection of the parameter levels (concentration (ppm), dose (mg), and time (min)), a number of tests were carried out. Table 2 presents the outcomes of the response operations and statistical plan.

**Table 2:** Experiments employing a Box-Behnken factorial design and three important operational parameters were conducted to optimize the percentage of phenol removed (%) from an aqueous solution by using modified fiber.

Trail	Conc. (ppm)	Dose (mg)	Time (min)	phenol removal percent (%) using control		phenol removal percent (%)using 0.25 modified fiber		phenol removal percent (%)using 0.5 modified fiber	
				Measured	Predicted	Measured	Predicted	Measured	Predicted
1	300	0.5	60	46.11	48.12718	55.76	57.845	61.93	70.32125
2	300	2	30	56.39	58.1198	75.21	79.0922	84.76	89.3372
3	500	2	60	50.09	50.5298	48.76	55.2032	74.59	77.5942
4	300	2	30	56.39	58.1198	75.21	79.0922	84.76	89.3372
5	500	0.5	30	29.96	40.22968	25.33	44.4455	46.9	68.72125
6	300	0.5	0	0	0	0	0	0	0
7	500	2	0	0	4.7498	0	7.6412	0	0
8	100	5	30	60.34	57.3447	82.57	77.167	99.29	94.1049
9	300	2	30	56.39	58.1198	75.21	79.0922	84.76	89.3372
10	100	0.5	30	47.29	44.94968	61.49	60.3055	81.08	76.78125
11	500	5	30	40.24	46.1447	52.64	62.567	64.37	78.3049
12	300	5	0	0	2.3897	0	6.413	0	3.7739
13	300	5	60	59.52	63.0197	77.21	85.421	79.89	89.7959
14	100	2	60	63.39	64.6098	85.21	88.6432	98.76	100
15	100	2	0	0	4.4298	0	5.0812	0	10.2402

The ANOVA technique was used to determine the significance of the coefficients and the adequacy of the suggested models. The *p* value was used to evaluate if each coefficient was significant or not. A model term's coefficient is insignificant if the *p* value is higher than 0.05. Table 2 is a list of the final ANOVA for every model term. The first order effects for all variables (X_1_, X_2_, and X_3_) of PAN and PAN/2.5 GO are significant, according to the *p* values; however, for PAN/5 GO nanofibers, the variable X_2_ is not significant. The remaining terms in the model, whose probability values exceed 0.05, are not significant. The insignificant model terms were eliminated to simplify this model. The determination coefficient (R^2^) was used to evaluate how well the models fit the empirical data. The capacity of the generated models to satisfactorily explain the behavior of the system within the examined series of operating factors is indicated by a high values of R^2^ 0.989 for phenol percent removal (%).

The findings showed that the removal percent (%) of phenol varied depending on the values of the three variables, which was caused by the impact of various variable levels on the removal percent (%) of phenol. According to the study and Table 3, test experiments 6 and 14 for PAN, PAN/2.5 GO, and PAN/5 GO yielded the lowest and highest removal amount of removal percent (%) of phenol by the BBD method, which was approximately 0, 0, 0 and 63.39, 85.21, 98.76%, PAN, PAN/2.5 GO, and PAN/5 GO, respectively.

**Table 3:** ANOVA results for removal percent (%) of phenol.

Source	PAN		PAN/2.5 GO		PAN/5 GO	
	*p* value	Significant or insignificant model terms	*p* value	Significant or insignificant model terms	*p* value	Significant or insignificant model terms
Model	0.001702	Significant	0.004447	Significant	0.005151	Significant
X_1_	0.008480	Significant	0.003105	Significant	0.017060	Significant
X_2_	0.030272	Significant	0.036576	Significant	0.099851	
X_3_	0.000011	Significant	0.013404	Significant	0.000080	Significant
X_1_^2^	0.078242		0.036576	Significant	0.702047	
X_2_^2^	0.014541	Significant	0.013404	Significant	0.060782	
X_3_^2^	0.000155	Significant	0.014598	Significant	0.000477	Significant
X_1_X_2_	0.480387		0.000030	Significant	0.688008	
X_1_X_3_	0.185781		0.000240	Significant	0.255157	
X_2_X_3_	0.232549		0.927899		0.540006	

Furthermore, the model's high accuracy in estimating the removal percent (%) of phenol was demonstrated by a slight deviation between the experiment and the projected removal percent (%) of phenol^33^. So as to determine the optimal model for the association between the response variables (the elimination percent (%) of phenol) and the major variables, a polynomial analysis and a second-order quadratic model were employed. The experimental data were subjected to multiple regression analysis, yielding the second-order polynomial equation (Eq. 1). This could provide an explanation for the phenol elimination percentage (%) for PAN, PAN/2.5 GO, and PAN/5 GO. The model's second order parameters were x_1_^2^, x_2_^2^, and x_3_^2^, together with the interaction parameters X_1_X_2_, X_1_X_3_, and X_2_X_3_.The linear parameters in Eq. (1) were X_1_, X_2_, and X_3_

1 $$\begin{aligned} {\text{Y}}_{{{\text{control}}}} = & - {17}.{5178} + 0.0{\text{682X}}_{{1}} + {12}.{2}0{2}0{\text{X}}_{{2}} + {2}.{514}0{\text{X}}_{{3}} - 0.000{\text{1X}}_{{1}}^{{2}} \\ & - {1}.{\text{8841X}}_{{2}}^{{2}} - 0.0{\text{256X}}_{{3}}^{{2}} - 0.00{\text{36X}}_{{1}} {\text{X}}_{{2}} - 0.000{\text{6X}}_{{1}} {\text{X}}_{{3}} + 0.0{\text{425X}}_{{2}} {\text{X}}_{{3}} \\ {\text{Y}}_{{0.{\text{25GO}}/{\text{PAN}}}} = & - {28}.{476}0 + 0.{\text{1258X1}} + {17}.{\text{1858X2}} + {3}.{\text{5233X3}} - 0.000{\text{2X12}} \\ & - {2}.{\text{8636X22}} - \, 0.0{\text{355X32}} + 0.000{\text{7X1X2}} - 0.00{\text{15X1 X3}} + 0.0{\text{747X2 X3}} \\ {\text{Y}}_{{0.{\text{5GO}}/{\text{PAN}}}} = & - {14}.{8}0{76} + 0.0{12}0{\text{X1}} + {17}.{\text{7323X2}} + {4}.{\text{1387X3}} + 0{\text{X12}} - {2}.{\text{6892X22}} \\ & - 0.0{\text{438X32}} - 0.00{\text{43X1 X2}} - 0.00{1}0{\text{X1 X3}} + 0.0{\text{446X2 X3}} \\ \end{aligned}$$

where, Y is the response (phenol removal percentage (%)) and X1,X2and X3 are the coded values of the test variables, e.g., concentration (ppm), dose (mg), and time(min),respectively.

The model is regularly insignificant and unsuccessful in predicting test results, with a value of "prob > F"more than 0.05. The *p* values for PAN, PAN/2.5 GO, and PAN/5 GO in this model were 0.001702, 0.004447, and 0.005151, respectively, suggesting that the model was statistically significant.

**Figure 3:**
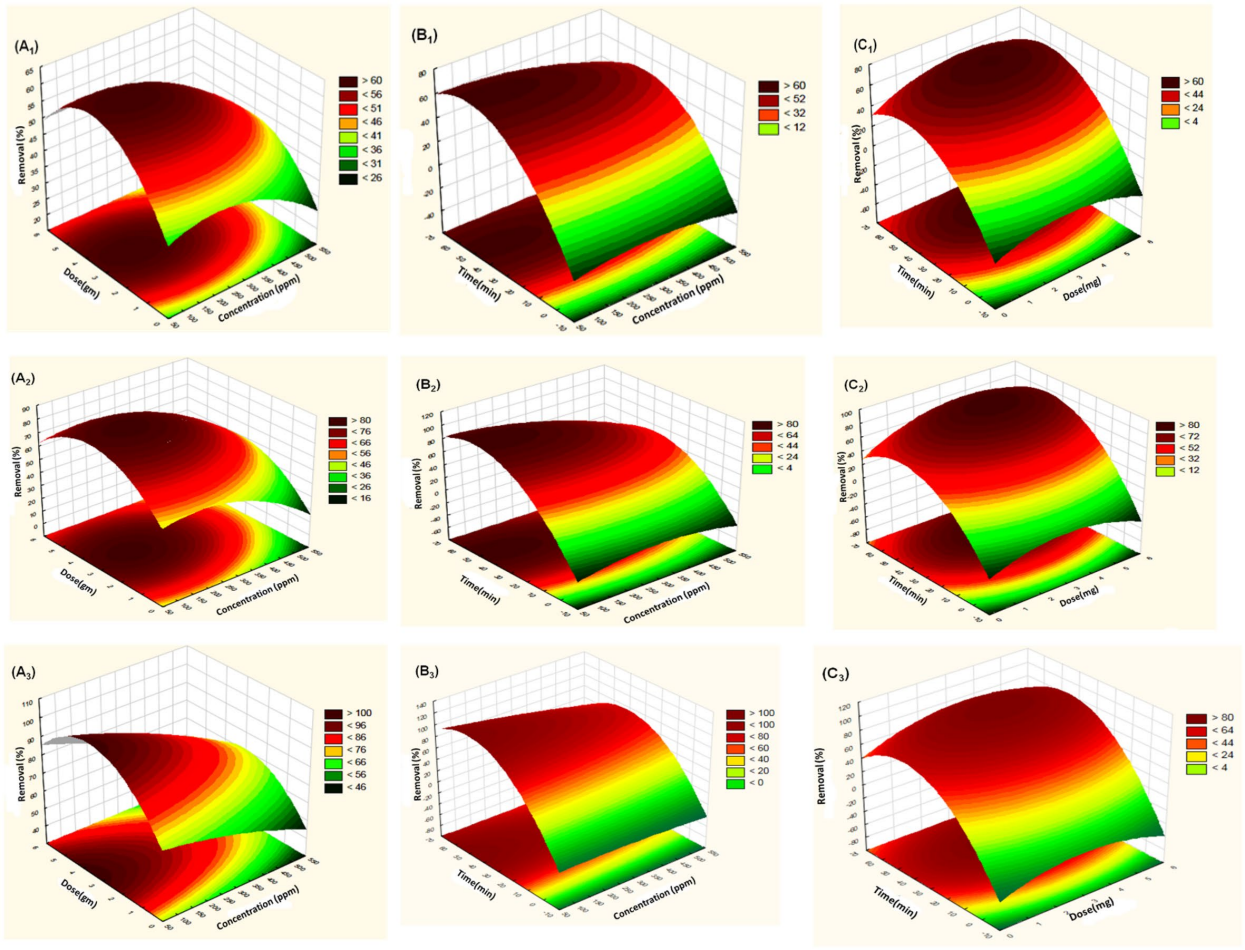
Response surface methodology of removal percent (%) of phenol using PAN, PAN/2.5 GO fiber, and PAN/5 GO fiber: (**A**) effect of Dose(mg) / concentration (ppm), (**B**) effect of time (min)/concentration (ppm), (**C**) effect of time(min) / Dose(mg), (1, 2 and 3) refer to (PAN, PAN/2.5 GO, and PAN/5 GO) respectively.

As seen in Fig. 3B1,B2, and B3, the interaction between time and concentration produced the largest response. The simultaneous effects of concentration and dose, agitation time, and elimination percent (%) of phenol using PAN, PAN/2.5 GO, and PAN/5 GO, respectively, are shown in Fig. 3A1,B1, A2,B2, and A3,B3, which reduced when the concentration amplified from 100 to 500 ppm, and the highest phenol removal percentage (%) for all fiber types was achieved with 300 ppm of phenol.

Conversely, Fig. 3(A1,C1, A2,C2, and A3,C3 illustrates how dose, concentration, and duration affect the percentage of phenol removed using PAN, PAN/2.5 GO, and PAN/5 GO, respectively. The phenol solution contains a fixed amount of phenol molecules, and the surface area of the adsorbent grew as the dose increased from 0.5 to 5 mg due to the increase in PAN, PAN/2.5 GO, and PAN/5 GO. This is because the active sites are available over the surface of the adsorbent.

The effects of duration and concentration, dose, and PAN, PAN/2.5 GO, and PAN/5 GO on the elimination percent (%) of phenol are shown in Fig. 3B1, C1, B2, C2, and B3, C3. Which grew when the agitation period was extended from 0 to 60 min because, during that time, the adsorbent's networks spread quickly in the beginning and reached equilibrium after 60 min. Therefore, it was considerably simpler for the removal percent (%) of phenol to first enter PAN, PAN/2.5 GO, and PAN/5 GO and mix with the adsorption sites^34,35^. However, as time went on, the number of active groups on the adsorbent's surface decreased, while the percentage of phenol removed (%) remained constant.

### Batch experiments

#### Effect of contact time and initial phenol concentrations

**Figure 4:**
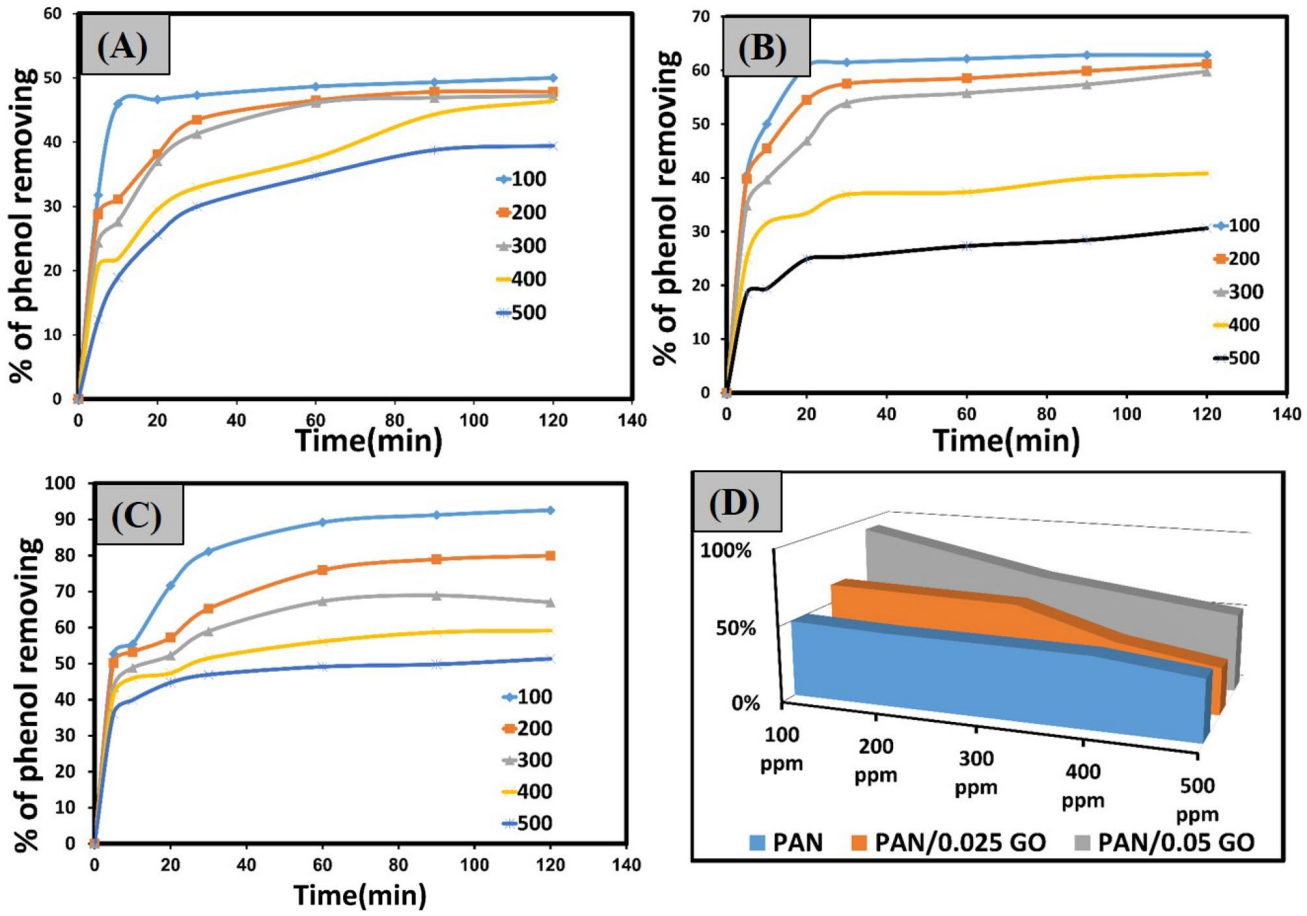
(**a**, **b**, and **c**): The effect of contact time on the phenol removal efficiency % at initial concentrations of phenol from 100 to 500 ppm for PAN nanofiber, PAN/2.5 GO, and PAN/5 GO, respectively. (**d**) Represent 3D graph for the maximum removal efficiency % of phenol for PAN nanofiber, PAN/2.5 GO, and PAN/5 GO at initial concentrations of phenol (100-500ppm).

The contact time effect on phenol removal efficiency was studied from 0 to 120 min at pH 7, at diverse phenol concentrations (100–500 ppm), and adsorbent dosage for PAN, PAN/2.5 GO, and PAN/5 GO was 5 mg. The shaking speed was specified to 200 rpm, and the experiment was took place at room temperature. Figure 4A–C illustrates how increasing the contact duration led to a gradual improvement in the phenol removal efficiency and a corresponding decrease in the hydrated layer's resistance at the boundary layer. However, after 120 min, the equilibrium point was reached when the adsorption rate equaled the desorption rate and there was no further phenol removal. Additionally, at the equilibrium, the adsorbent active site decreased, causing the plateau to be reached^36^. Furthermore, at the same contact time the phenol elimination efficiency of PAN/GO nanofibers was greater than that of pure PAN nanofibers. As demonstrated in Fig. 4D, the phenol removal efficiency for PAN, PAN/2.5 GO, and PAN/5 GO decreased as the initial concentration increased from 100 to 500 ppm, from (50 to 39.42%), (62.838 to 30.61%), and (92.568% to 50%), respectively. This was because at higher concentrations, the accessible active sites decreased as a result of phenol ion competition for these sites^37^.

#### Effect of nanofibers dosage

**Figure 5:**
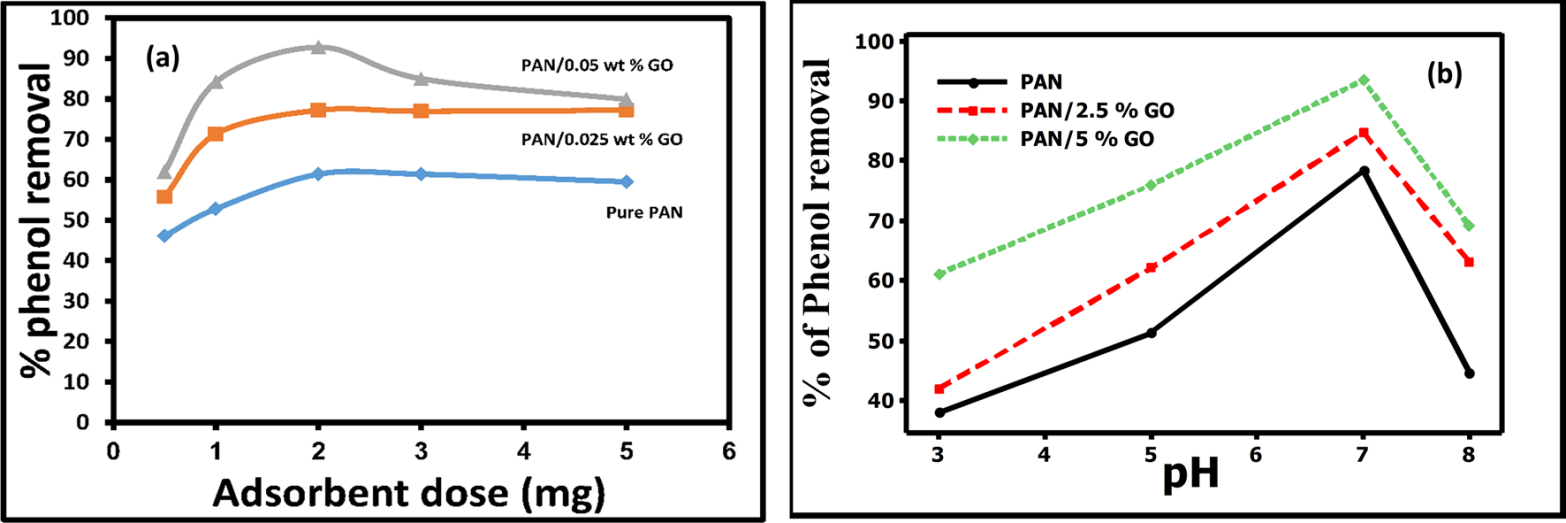
(**a**) Effect of the nanofibers dosage on the adsorption of phenol, (**b**) Effect of pH on the adsorption of phenol.

The experiment's dosage of nanofibers was varied (between 0.5 and 5 mg), while all other variables remained constant (Fig. 5a). It was found that increasing the dosage of the adsorbent until it reached 2 mg caused an increase in the removal percentage. Maximum removals for pure PAN, PAN/2.5GO, and PAN/5GO were 61.3941, 77.2118, and 92.76139%, respectively. At dosages above 2 mg, the removal percentage starts to slightly decline and then stabilizes at 5 mg of the adsorbent. The increase in active adsorption sites until the ideal dosage was reached was the explanation for this finding. However, because the active adsorption sites gathered or overlapped at dosages higher than the recommended amount, removal efficiency stayed constant above the optimal level^38–40^.

#### Effect of pH

The pH of an aqueous solution is an important variable in the adsorption of ions. It influences the adsorbate chemistry in water as well as the surface binding sites of the adsorbent. While all other parameters, including contact duration, adsorbent dosage, and starting concentration, stayed constant at 140 min, 2 mg, and 100 ppm, respectively, the degree of phenol removal was evaluated at pH values ranging from 3 to 8 Fig. 5b. The optimum pH for phenol removal was 7 for all adsorbent nanofiber mats, with maximum removal percentages of (78.3%, 84.7%, and 93.4%) for pure PAN, PAN/2.5 GO, and PAN/5 GO, respectively. Because of the difference in solution ionic chemistry and the surface charge of the adsorbent and phenol, the efficiency of phenol removal decreases above pH 7, and this result is similar to that obtained by using carbon nanofiber (CNF) for phenol adsorption^41^. Table 4 demonstrates that our adsorption ratios are higher than those reported in previous literature.

**Table 4:** The characteristics and phenol adsorption capacity onto newly investigated nanofiber mat adsorbents.

Adsorbent (nanofibers mats)	Time (min)	pH	Adsorption capacity (mg/g)	Reference
CNFs	710	7	0.842	^39^
CNF–Fe2O3	710	7	1.684	^39^
M-ZnO/PVA/Alg/CS	200	5	10.03	^42^
P(3HB-co-3HHx)	270	4	59.047	^43^
Pure PAN	120	7	57.4	This work
PAN/2.5% GO	120	7	66.18	This work
PAN/5% GO	120	7	69.7	This work

CNF: Carbon nanofiber, PVA: Polyvinyle alchol, Alg: Alginate, CS: Chitosan nanofiber, P(3HB-co-3HHx): poly(3- hydroxybutyrate-co-3-hydroxyhexanoate).

#### Adsorption isotherms

Adsorption isotherms offer details on the capacity for adsorption and the interactions between molecules of the adsorbate and the adsorbent. In the current study, phenol adsorption testing is conducted using the Langmuir and Freundlich models. The Langmuir isotherm is relevant to homogeneous surfaces with equal adsorption sites affinity, whereas the Freundlich model is appropriate to heterogeneous adsorption sites^44^.

**Figure 6:**
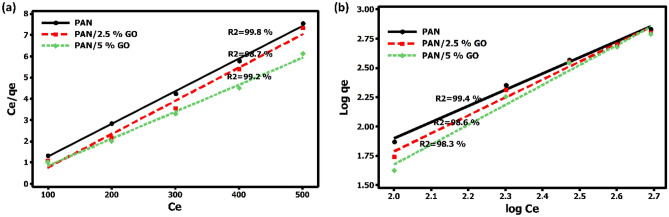
Linear isotherm models (**a**) Langmuir and (**b**) Freundlich.

Figure 6 depicts isotherm models, and Table 5 shows the calculated parameters derived from these two models. As shown in Fig. 6 for phenol adsorption, both adsorption models fit well. A strong correlation coefficient indicates that the Langmuir model fit the adsorption better, suggesting monolayer phenol adsorption on the surface of nanofiber mats. The adsorption capabilities of pure PAN, PAN/2.5 GO, and PAN/2.5 GO were found to be 57.4, 66.18, and 69.3 mg/g, respectively, based on the Langmuir isotherm model. The adsorption of phenol ions from water is significantly impacted by the impregnation of PAN with GO. Because GO has a larger surface area and more adsorption sites, phenol ions were strongly adsorbed to the PAN-GO surfaces. All of the nanofiber mats had Freundlich constants (n) less than 1, suggesting that phenol adsorption was advantageous on their surface. Because of their strong affinity and adsorption capacities, pure PAN and PAN/GO mats may be effective adsorbents for phenol removal %, according to findings of the isotherm investigation.

**Table 5:** Parameters of linear Langmuir and Freundlich isotherm models for phenol adsorption onto PAN, and PAN/GO nanofibers composites mats.

Adsorbent	Langmuir	Freundlich				
	q max (mg/g)	K_L_	R^2^	K_f_	1/n	R^2^
Pure PAN	57.4	0.064	99.8	0.133	1.386	99.4
PAN/2.5% GO	66.18	0.033	98.7	0.0517	1.536	98.6
PAN/5% GO	69.7	0.018	99.2	0.0187	1.701	98.3

#### Kinetic studies

**Figure 7:**
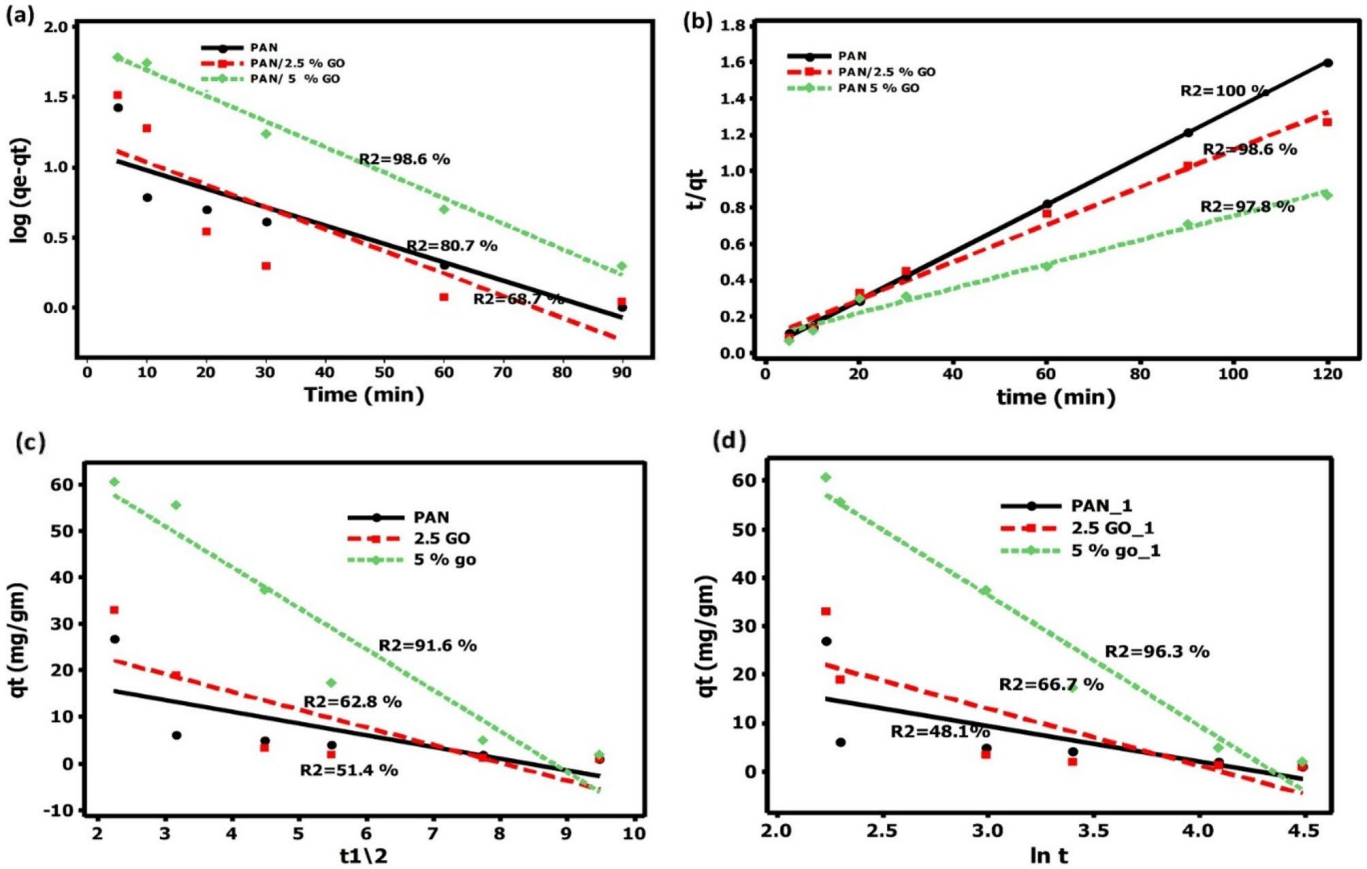
(**a**) Pseudo-first-order kinetic plot (**b**) Pseudo-second-order kinetic plot for phenol adsorption, (**c**) intra-particle diffusion model, and (**d**) Elovich model.

The investigation focused on the kinetics of the adsorption process, employing four kinetic models: the pseudo 1st order—pseudo 2nd order—intra-particle diffusion, in addition Elovich kinetic models. The linear plots corresponding to these models are presented in Table 5. The degree of agreement between the experimental data and the calculated values of the model was assessed using the correlation coefficient (R^2^). This coefficient provides a measure of the model's ability to accurately describe the kinetics of adsorption. Figure 7A–C demonstrates the plots that were produced using the equations for the linear and nonlinear kinetic model. In addition, Table 6 provided an overview of the four models' estimated parameters and corresponding R^2^.

**Table 6:** Kinetic parameters for the adsorbents (PAN, PAN/2.5%, PAN/5%GO).

Model/parameter	PAN	PAN/2.5%GO	PAN/5%GO
Pseudo first order			
q_e exp_	75.8	112.8	148.3
q_e cal_	12.97	15.66	75.33
K_1_	0.030	0.036	0.041
R^2^	0.807	0.687	0.986
Pseudo second order			
q_e exp_	75.8	112.8	148.3
q_e cal_	76.3	100	151.5
K_1_	0.6	0.11	0.051
R^2^	1	0.986	0.978
Intra-particle			
diffusion C	− 183.7	− 191.5	− 181.6
K_diff_ (mg/g.min^1/2^)	92.13	89.14	85.52
R^2^	0.98	0.975	0.97
Elovich model			
β	0.003	0.038	0.004
α	58.33	31.61	27.37
R^2^	0.99	0.994	0.99

The findings depicted in Table 6 demonstrate that the pseudo second-order model and Elovich model provided more suitable description of the kinetic process of phenol adsorption on all the nanofiber mats examined, in comparison to alternative models. It suggests that the pseudo-second-order kinetic mode more accurately describes the sorption of phenol onto PAN nanofiber mats. This suggests that the rate-limiting phase for phenol onto nanofiber mats is chemisorption, in which valence forces are activated through electron sharing or exchange between the hydroxyl groups of the fibres and phenols. Moreover, the rise in the concentration of GO impregnated into the fiber led to an enhancement in the adsorption rate and this result similar to (^45^, and ^46^). Furthermore, Tshemese and their coworker have demonstrated that the adsorption of phenol on Exfoliated Graphite (EG) adheres to the pseudo-second order rate equation, as evidenced by regression coefficients exceeding 0.99.

The Elovich model (Fig. 7d) was employed as non linear kinetic model to describe the second-order kinetics. The parameters obtained from this model are presented in Table 6. The uptake speed constants (α) exhibited higher values compared to the desorption speed constants (β). The findings of this study underscore and validate the suitability of the adsorbents for the removal of micropollutants. This result similar to that in (^47^). In this study, it was observed that the Elovich kinetic models (with a coefficient of determination, R^2^, greater than 0.94) and the Langmuir isotherm models (with an R^2^ greater than 0.98) provided the most accurate representation of the experimental data. The specific surface area of graphene oxide (GO) is significantly high, and it possesses various oxygen functional groups.

Neither the pseudo first-order equation nor the intra-particle diffusion equation provided a satisfactory representation of the experimental data. This observation aligns with the regression coefficient value being relatively low, and the presence of discrepancies between the fitted qe and experimental values.

#### Thermodynamic study

The tests were accomplished at various temperatures (30–50 °C) and phenol concentration (100–500 ppm) with constant pH, dose, and equilibrium time using PAN, PAN/2.5% GO and PAN/5% GO. The outcomes illustrated a decrease in phenol removal percent with increase temperature signifying that the adsorption of phenol on fiber and modified fiber more easily happened at room temperature^48,49^. The values of ΔS^°^ and ΔH^°^ are calculated from the slopes and intercepts of the linear plots of lnb versus 1/T, ΔG° is obtained using (Eq. 1). The thermodynamic parameters of phenol adsorbed by fiber and modified fiber are listed in (Table 7) The negative values of ΔH^◦^ recommend that the interaction of phenol adsorbed by fiber and modified fiber is exothermic, which is supported by the decrease adsorption of phenol adsorbed by fiber and modified fiber with a rise in temperature^50^. Adsorption was facilitated by lowering the temperature, and it exhibited enthalpy-driven adsorption-type behavior. Adsorption was not entropy-driven, as evidenced by ∆S° < 0 for phenol adsorbed by fiber and modified fiber, which decreased the level of unpredictability in the adsorption process. If ∆G < 0, then the adsorption process was spontaneous^49^.

**Table 7:** Adsorption thermodynamic parameters for phenol adsorption on fiber and modified fiber.

Materials	T (°K)	Δ G (KJ mol^−1^)	Δ S (KJ mol^−1^ K^−1^)	Δ H (KJ mol^−1^)
Pure PAN	303	− 2.79299	− 0.47315	− 139.817
	313	− 9.94551		
	323	− 12.1519		
PAN/2.5% GO	303	− 2.0597	− 0.86216	− 260.245
	313	− 16.5622		
	323	− 14.7576		
PAN/5% GO	303	− 8.28622	− 0.22024	− 56.211
	313	− 17.5381		
	323	− 12.3857		

## Conclusion

The present study involved the synthesis of PAN, PAN/2.5% GO, and PAN/5% GO nanofibers through the utilisation of the electrospinning technique. The incorporation of graphene oxide (GO) renders the nanofiber appropriate for the elimination of phenol from aqueous solutions. The nanofibers were effectively synthesised and characterised using scanning electron microscopy (SEM) and Fourier-transform infrared spectroscopy (FTIR). The Response Surface Method (RSM) was employed to facilitate the experimental design process. The statistical analysis of variance (ANOVA) demonstrated that the concentration of phenol, pH level, the interaction between the concentration of phenol and the dose of the adsorbent all have a noteworthy effect on the removal of phenol. The findings suggest that the adsorption process can be accurately described by the second-order model, Elovich model, and all the adsorbents conform to the Langmuir isotherm model. PAN, PAN/2.5 GO, and PAN/5 GO were shown to have maximal monolayer adsorption capacities of 57.4, 66.18, and 69.7 mg/g, respectively. The thermodynamic analysis showed that an exothermic process was involved in the phenol's adsorption onto all adsorbents.

### Supplementary Information

Supplementary Information.

## Data Availability

This article contains all of the data created or analysed during this investigation.
