# Supplementary material for: Thermodynamic and kinetic analysis of the response surface method for phenol removal from aqueous solution using graphene oxide-polyacrylonitrile nanofiber mats
Source: Sci Rep. 2024 Feb 12;14:3531. doi: 10.1038/s41598-024-53572-5 (PMC10861576; doi:10.1038/s41598-024-53572-5)
Supplement: Supplementary file 1 — Supplementary Information. [file 41598_2024_53572_MOESM1_ESM.docx]

**Thermodynamic and Kinetic Analysis of the Response Surface Method for Phenol Removal from Aqueous Solution Using Graphene Oxide-Polyacrylonitrile Nanofiber Mats**

**Bassant Yousri Eweida ^1^, Asmaa M. Abd El-Aziz*^2^, Azza El-Maghraby^2^, Eman Serag*^3^**

^1^Modeling and Simulation Research Department, Advanced Technology and New Materials Research Institute, City of Scientific Research and Technological Applications, Alexandria, Egypt, [basant177@yahoo.com](mailto:basant177@yahoo.com)

^2^Fabrication Technology Research Department, Advanced Technology and New Materials Institute, City of Scientific Research and Technological Applications (SRTA-City), Borg El-Arab, Alexandria, Egypt, email: [maghrabyazza@yahoo.com](mailto:maghrabyazza@yahoo.com), [chemist_asmaa25@yahoo.com](mailto:chemist_asmaa25@yahoo.com), [aabdelaziz@srtacity.sci.eg](mailto:aabdelaziz@srtacity.sci.eg)

^3^Marine Pollution Department, Environmental Division, National Institute of Oceanography and Fisheries (NIOF), Kayet Bey, Elanfoushy, Alexandria, Egypt, [d.emanserag@yahoo.com](mailto:d.emanserag@yahoo.com).

**Methods**

- **Characterization of the prepared samples**

### Fourier Transmission - IR Spectrophotometer (FTIR -8400S, Shimadzu, Japan) with a resolution of 2 cm^-1^ is used for measure the modify in the functional groups before and after functionalization of the nanofibers with GO-NP. Crystals of dry potassium bromide (KBr) (20 mg) were mixed with the samples (5 mg) at 27ºc. The range of 4000-350 cm^−1^ was used to record the spectrum. The nanofiber precursor samples were inspected under a scanning electron microscope (SEM) equipped with an Electron Dispersive X-Ray unit (EDX) from Japan (JEOL JSM 6360LA).

### **Experimental design**

The bellow quadratic equation was accustomed to calculate the correlation between the response and independent variables after the experiments were conducted (**Eq.S1**):

**Y=β0+∑βixi + ∑ βii xi^2^ + ∑βijxixj Eq.S1**

In the given equation, Y represents the predicted response, β0 represents the intercept term, βi represents the linear effect, βii represents the square effect, and βij represents the interaction effect.

The acceptance or rejection of model elements is contingent upon the value of F and the likelihood of error (P) at a 95% confidence level. Using Statistic software, the outcomes of the BBD were examined using the ANOVA and F test ^18^. The two elements' combined influence resulted in the acquisition of 3D plots and the corresponding contour plots, while other factors are modifiable by default. Because of this, BBD data be able to obtained in three-dimensional presentations that include contours. This will make it easier to research how variables interact with the replies simultaneously ^15^.

- **Phenol removal tests**

$\boldsymbol{q}_{\boldsymbol{e}}\boldsymbol{=}\frac{\boldsymbol{C}_{\boldsymbol{O-}}\boldsymbol{C}_{\boldsymbol{t}}}{\boldsymbol{m}}\boldsymbol{V}$ **Eq.S2**

The variable qe (mg/g) represents the quantity of metal adsorbed per gramme of the adsorbent at equilibrium. Co denotes the initial concentration of phenol in the liquid phase (mg/L), while Ct represents the concentration of phenol in the liquid phase at time t (mg/L). The variables m and V correspond to the amount of adsorbent (mg) and the volume of the solution (L), respectively.

### **Adsorption Isotherms**

Based on the assumption of uniform surface adsorption, the Langmuir isotherm model ^20^. The Freundlich isotherm model assumes the presence of heterogeneous non-uniform heat distribution on the surface ^21^. It is possible to express the Langmuir and Freundlich models through the use of Eqs.

$\frac{\boldsymbol{C}_{\boldsymbol{e}}}{\boldsymbol{q}_{\boldsymbol{e}}}\boldsymbol{=}\frac{\boldsymbol{1}}{\boldsymbol{Q}_{\boldsymbol{max}}\boldsymbol{k}_{\boldsymbol{l}}}\boldsymbol{+}\frac{\boldsymbol{C}_{\boldsymbol{e}}}{\boldsymbol{Q}_{\boldsymbol{max}}}$  **Eq. S3**

$\boldsymbol{Log}\boldsymbol{q}_{\boldsymbol{e}}\boldsymbol{=}\log\boldsymbol{k}_{\boldsymbol{f}}\boldsymbol{+(}\frac{\boldsymbol{1}}{\boldsymbol{n}}\boldsymbol{)}\log\boldsymbol{C}_{\boldsymbol{e}}$ **Eq. S4**

### **Kinetics Studies**

The linear kinetic pseudo-first-order, pseudo-second-order, nonlinear kinetic intra-particle diffusion, and Elovich models, which can be expressed in linear forms as follows:

$\boldsymbol{Ln}\left( \boldsymbol{q}_{\boldsymbol{e}}\boldsymbol{-}\boldsymbol{q}_{\boldsymbol{t}} \right)\boldsymbol{=Ln(}\boldsymbol{q}_{\boldsymbol{e}}\boldsymbol{-}\boldsymbol{k}_{\boldsymbol{1}}\boldsymbol{t)}$ **Eq. S5**

$\frac{\boldsymbol{t}}{\boldsymbol{q}_{\boldsymbol{t}}}\boldsymbol{=}\frac{\boldsymbol{1}}{{\boldsymbol{k}_{\boldsymbol{2}}\boldsymbol{q}}_{\boldsymbol{e}}^{\boldsymbol{2}}}\boldsymbol{+}\frac{\boldsymbol{1}}{\boldsymbol{q}_{\boldsymbol{e}}}\boldsymbol{t}$ **Eq. S6**

$\boldsymbol{q}_{\boldsymbol{t}}\boldsymbol{=}\boldsymbol{K}_{\boldsymbol{dif}}\boldsymbol{t}^{\boldsymbol{1/2}}\boldsymbol{+C}$ **Eq. S7**

$\boldsymbol{q}_{\boldsymbol{t}}\boldsymbol{=}\frac{\boldsymbol{1}}{\boldsymbol{\beta}}\boldsymbol{ln}\left( \boldsymbol{\alpha\beta} \right)\boldsymbol{+}\frac{\boldsymbol{1}}{\boldsymbol{\beta}}\boldsymbol{ln}\left( \boldsymbol{t} \right)$ **Eq. S8**

Qe is the equilibrium adsorption capacity in mg/g, and qt is the amount of phenol adsorbed at a specific time t, measured in mg/g. k_1_ and k_2_ stand for the pseudo-first-order and pseudo-second-order rate constants, correspondingly. Here are some definitions of the variables used in models of nonlinear intra-particle diffusion kinetics: Within the non-linear Elovich model, α stands for the initial adsorption rate (in mg/(g.min)) and β for the desorption constant (in g/mg), while K_dif_ is the intra-particle diffusion rate (in mg/(g.min^1/2^)) and C is the intercept.

- - - **Adsorption thermodynamics model**

The relationship between the Gibbs free energy change of the adsorption process and the equilibrium constant can be expressed by the following equation:

**ΔGº = − RT ln b Eq. S9**

The given equation can be summarised as follows: ΔG◦ represents the standard free energy change in KJ per mole, T stands for absolute temperature in Kelvin, R is the ideal gas constant (8.3145 J/mol·K), and b is an equilibrium constant and Langmuir constant obtained**.** ^22^

Here is the equation that describes the relationship between the change in Gibbs free energy, the change in entropy, and the heat of adsorption at a constant temperature, as stated in thermodynamics:

$\boldsymbol{\Delta}\boldsymbol{G}^{\boldsymbol{0}}\boldsymbol{=}\boldsymbol{\Delta}\boldsymbol{H}^{\boldsymbol{0}}\boldsymbol{-} \boldsymbol{T\Delta}\boldsymbol{S}^{\boldsymbol{0}}$ **Eq. S10**

**Ln b= (ΔSº/R) – (ΔHº/RT) Eq. S11**

The values of ΔH◦ and ΔS◦, which represent the change in enthalpy (KJ mol^−1^) and entropy (KJ mol^−1^ K^−1^), can be determined by plotting (ln b) against (1/T).
